# Lung Cancer in a Patient With Pulmonary Artery Sling: A Case Report

**DOI:** 10.3389/fsurg.2022.849183

**Published:** 2022-02-23

**Authors:** Jiahui Mi, Xun Wang, Xiao Li, Yanguo Liu, Guanchao Jiang, Feng Yang

**Affiliations:** Department of Thoracic Surgery, Peking University People's Hospital, Beijing, China

**Keywords:** pulmonary artery sling, lung cancer, lobectomy, perioperative risk, prognosis

## Abstract

**Introduction:**

Pulmonary artery sling (PAS) is a rare congenital vascular anomaly that results when the left pulmonary artery arises from the right pulmonary artery. There is little relevant literature on lobectomy for the treatment of lung cancer in patients with PAS, and the prognosis is unknown.

**Case Description:**

A 54-year-old asymptomatic man was found to have a nodule on the left lower lobe of the lung, which measured 2.5 cm. The patient also had PAS. Three-dimensional computed tomography angiography confirmed that the left pulmonary artery arose from the right pulmonary artery and passed between the main trachea and the esophagus toward the left thorax. No obvious contraindication was found in the preoperative examination, and the patient successfully underwent lobectomy of the left lower lobe by video-assisted thoracoscopic surgery. Histological examination of the lesion revealed invasive adenocarcinoma. The postoperative course was uneventful, and no complications occurred in the subsequent 3 years of follow-up.

**Conclusions:**

Lobectomy in a lung cancer patient with PAS did not increase perioperative risk and had no significant effect on prognosis.

## Introduction

Pulmonary artery sling (PAS) was first recognized in 1897 by Glaevecke and Doehle (1). Most patients with PAS are diagnosed as infants, according to severe respiratory symptoms. There are very few reports about PAS in adulthood (2), and an extensive review of the literature found that there is only one report involving adult patients with PAS who undergo lung cancer surgery (3). Therefore, it is unknown whether the presence of PAS will increase perioperative risk or affect the prognosis in lung cancer patients. Herein, we present the detailed preoperative examination results and prognosis of an asymptomatic lung cancer patient with PAS.

## Case Report

In November 2018, a 54-year-old asymptomatic male with 20 pack-year smoking history received a health examination. A nodule on the left lower lobe, measuring 2.5 cm, and PAS were detected by chest computed tomography (CT) for the first time in his life. The patient had no respiratory symptoms or other illnesses and there was a history of pneumonia in his childhood, which had never relapsed after adolescence. No abnormal indicators were observed on physical examination, and the patient denied having lost weight in the past 6 months.

The patient's pulmonary function test results were normal. The forced vital capacity (FVC) was 3.73 L and forced expiratory volume in one second (FEV1) was 3.35 L. FVC percent predicted (FVC % pred), FEV1 percent predicted (FEV1 % pred), and FEV1/FVC ratio was 80.7, 90.0, and 89.3%, respectively. The 99mTc-macroaggregated albumin pulmonary perfusion showed that the left pulmonary perfusion accounted for 31.4% of the total pulmonary perfusion, and the left lower lobe perfusion accounted for 56.5% of the left pulmonary perfusion. In addition, we performed a whole-body bone scan and head magnetic resonance imaging (MRI) for the patient before surgery, and it was confirmed that the patient had no distant metastasis. The carcinoembryonic antigen was increased slightly to 5.02 ng/mL (reference value < 4.70 ng/mL). Routine blood examination, arterial blood gas tests and electrocardiogram showed no abnormalities.

**Figure 1:**
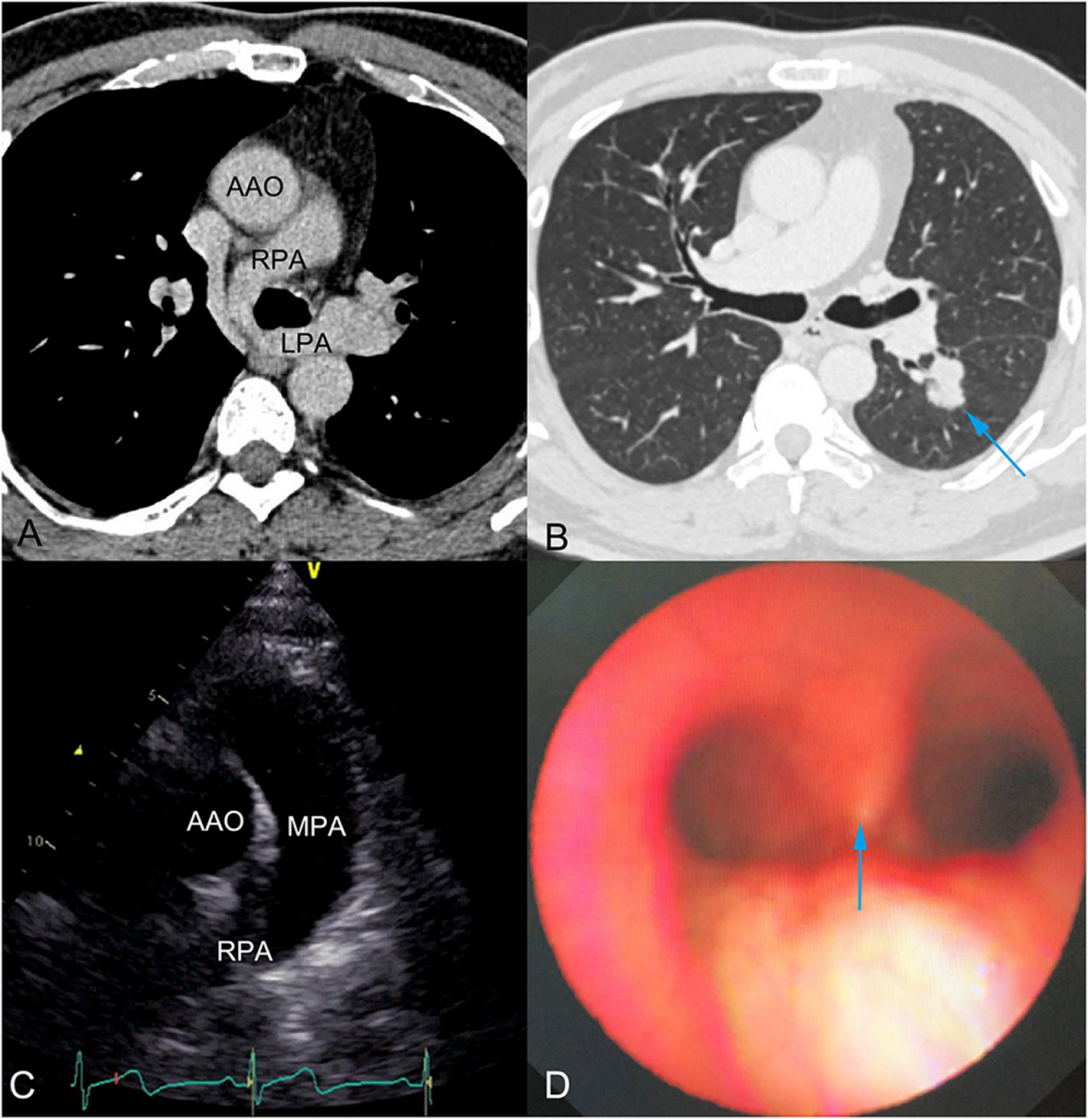
**(A)** Chest computed tomography (CT) showed pulmonary artery sling, which was observed for the first time during his life. **(B)** Preoperative chest CT showed a nodule of 2.5 cm diameter and indicated the possibility of malignancy (blue arrow). **(C)** Transthoracic echocardiography showed the main pulmonary artery had only one outflow tract to the right pulmonary artery. The beginning of the left pulmonary artery could not be detected, due to its deep position. **(D)** Bronchoscopy showed no obvious tracheal stenosis or bronchomalacia. Blue arrow indicates the tracheal carina. AAO, Ascending aorta; RPA, Right pulmonary artery; LPA, Left pulmonary artery; MPA, Main pulmonary artery.

**Figure 2:**
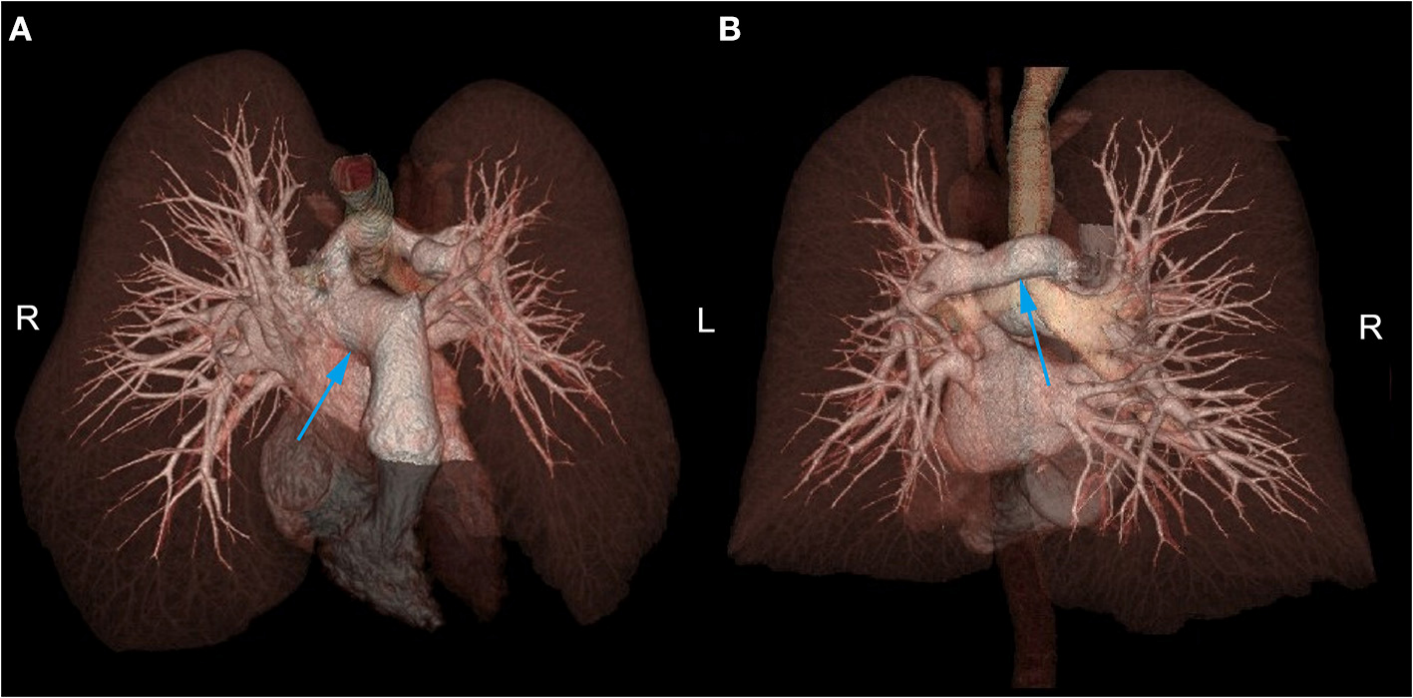
**(A)** In the anterior superior aspect, the left pulmonary artery was shown to arise from the right pulmonary artery. **(B)** In the posterior aspect, the left pulmonary artery was shown to pass between the main trachea and the esophagus toward the left thorax. R, Right side; L, Left side.

Chest CT showed PAS (Figure 1A) and a nodule on the left lower lobe, measuring 2.5 cm, which indicated the possibility of malignancy (Figure 1B). Transthoracic echocardiography confirmed that the main pulmonary artery had only one outflow tract to the right pulmonary artery (Figure 1C). However, we could not detect the beginning of the left pulmonary artery due to its deep position. In addition, neither obvious tracheal stenosis nor bronchomalacia was found by bronchoscopy (Figure 1D). Three-dimensional CT angiography was used to demonstrate that the left pulmonary artery arose from the right pulmonary artery and passed between the main trachea and the esophagus toward to the left thorax (Figure 2; Supplementary Video 1).

**Figure 3:**
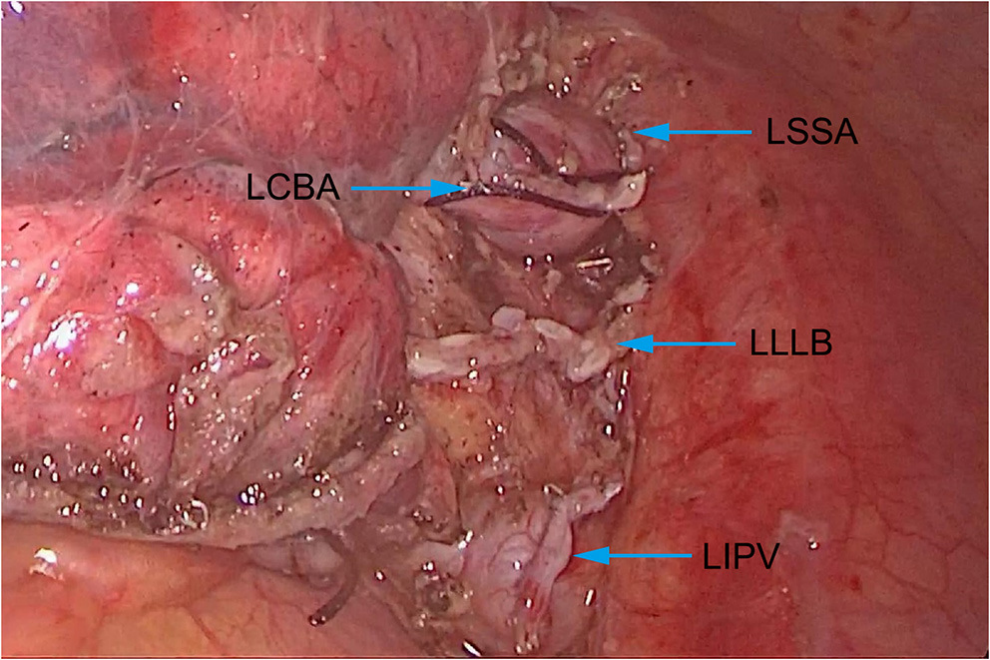
Anatomical structure of the artery, bronchus and vein of the left lower lobe. LSSA, Left superior segmental artery; LCBA, Left common basal artery; LLLB, Left lower lobe bronchus; LIPV, Left inferior pulmonary vein.

Through comprehensive preoperative assessments, no obvious contraindication was found in this patient. After multidisciplinary discussion and providing signed informed consent, it was decided to perform lobectomy on the patient by video-assisted thoracoscopic surgery. The patient was anesthetized with double-lumen endotracheal intubation. During the operation, it was observed that the left inferior lobe artery was adjacent to the posterior mediastinum and above the inferior lobe bronchus. We firstly performed wedge resection of the lesion during the operation. After intraoperative frozen section proved the malignant nature of the lesion, we continued to perform lobectomy on this patient. We excised the left inferior pulmonary vein, left superior segmental artery, left common basal artery and left lower lobe bronchus (Figure 3) and completely removed the left lower lobe. In addition, we performed systematic lymph node dissection for this patient, and the presence of PAS did not significantly increase the technical difficulties of lymph node dissection. The operation time was 195 min, and the total blood loss was 90 ml.

The patient's postoperative course was uneventful, and he was discharged 4 days after the operation. Pathological results showed the nodule to be invasive adenocarcinoma, visceral pleura invasion, and the pathology stage to be IB (pT2aN0M0). There was no adjuvant chemotherapy or any other postoperative treatment prescribed.

Three months later, the patient's subsequent visit showed FVC of 2.87 L and FEV1 of 2.50 L. The FVC % pred, FEV1 % pred, and FEV1/FVC ratio was 91.4, 88.7, and 87.1%, respectively. Chest CT demonstrated the postoperative performance of the left lower lobe, which had been subject to the resection, and showed no other abnormalities. The 99mTc-macroaggregated albumin injection demonstrated that the left pulmonary perfusion accounted for 18.0% of the total pulmonary perfusion. The patient remained symptom free during the subsequent 3 years of follow-up.

## Discussion

PAS is a rare congenital vascular anomaly in which the left pulmonary artery arises extrapericardially from the posterior aspect of the right pulmonary artery. The left pulmonary artery passes over the right main bronchus and courses between the main trachea and the esophagus to reach the left pulmonary hilum (4). Most patients with PAS are identified in infancy, following investigation of severe respiratory symptoms. Asymptomatic adult PAS cases are rare but usually have an excellent prognosis and require no surgical therapy (5, 6). Mammana et al. (3) reported a case of an adult lung cancer patient with PAS, who underwent lung cancer surgery and was treated with pulmonary artery reconstruction due to tumor invasion. However, whether the presence of PAS will increase perioperative risk or affect the prognosis is still unknown. We have described herein an adult asymptomatic patient with lung cancer and PAS, who underwent lobectomy without treatment of the PAS, and also reported the detailed perioperative examination results and prognosis.

In the developing embryo, if the left postbranchial vessel cannot connect to the left sixth branchial arch correctly and another connection is formed to the right sixth arch *via* postbranchial channels between the trachea and the esophagus, then PAS occurs (7). Clinically, PAS is divided into two types, each of which are further divided into two subtypes according to the position of the pulmonary artery, as follows: type I PAS being located at the T4-5 level, just above the carina (IA without and IB with right tracheal bronchus); and type II PAS being located at the T6-7 level, just above the low horizontal carina (4). Our case was type IA. In general, type IA does not change the main structure of the tracheobronchus nor the overall structure of either lung. However, the abnormal left pulmonary artery is often close to the junction of the main bronchus and the right pulmonary bronchus. Continued oppression will result in bronchomalacia, which may cause air trapping and hyperinflation of the right lung. These situations may lead to cardiopulmonary surgery.

Fortunately, none of the above was found in our patient by bronchoscope or pulmonary function examinations. The endotracheal intubation went smoothly, and single-lung ventilation did not affect the patient's vital signs during surgery. In addition, three-dimensional CT angiography clearly showed the distribution of arteries, veins and trachea, providing accurate anatomy information for the surgeons. All these factors provided favorable conditions for the lobectomy by video-assisted thoracoscopic surgery and ensured intraoperative safety.

This case also demonstrated the results of pulmonary perfusion and pulmonary function in a patient with PAS before and after left lower lobectomy. The preoperative pulmonary perfusion of this patient revealed a mildly asymmetric flow to the left lung (31.4%) and right lung (68.8%), which is similar to a previously reported case (8). After the left lower lobectomy, the predicted left pulmonary perfusion of the total pulmonary perfusion was 16.6% and the actual left pulmonary perfusion accounted for 18.0%. Although the FVC and FEV1 both went down after the lobectomy, the FVC % pred, FEV1 % pred and FEV1/FVC ratio were all within the normal range. Therefore, the pulmonary perfusion and pulmonary function of the remaining lobes were not affected after the left lower lobectomy in our patient with PAS.

## Conclusion

This is the first report of an adult asymptomatic patient with lung cancer and PAS who underwent lobectomy without treatment of the PAS. An extensive preoperative examination and evaluation ensured smooth progress of the operation. This patient remained symptom free during the 3 years of follow-up. Therefore, adult asymptomatic patients with PAS can undergo lung surgery safely and may require no treatment for PAS.

## Data Availability Statement

The original contributions presented in the study are included in the article/Supplementary Material, further inquiries can be directed to the corresponding author.

## Ethics Statement

Written informed consent was obtained from the individual(s) for the publication of any potentially identifiable images or data included in this article.

## Author Contributions

JM reviewed the literature and drafted the manuscript. XW and XL analyzed the data. YL and GJ provided technical support. FY reviewed the manuscript. All authors issued approval of the final version.

## Conflict of Interest

The authors declare that the research was conducted in the absence of any commercial or financial relationships that could be construed as a potential conflict of interest.

## Publisher's Note

All claims expressed in this article are solely those of the authors and do not necessarily represent those of their affiliated organizations, or those of the publisher, the editors and the reviewers. Any product that may be evaluated in this article, or claim that may be made by its manufacturer, is not guaranteed or endorsed by the publisher.

## Supplementary Material

Supplementary Video 1 Three-dimension reconstruction of computed tomography angiographic.
